# Human Beta-Defensin 3 Is Up-Regulated in Cutaneous Leprosy Type 1 Reactions

**DOI:** 10.1371/journal.pntd.0001869

**Published:** 2012-11-01

**Authors:** Anna L. Cogen, Stephen L. Walker, Chrissy H. Roberts, Deanna A. Hagge, Kapil D. Neupane, Saraswoti Khadge, Diana N. J. Lockwood

**Affiliations:** 1 Faculty of Infectious Tropical Diseases, Department of Clinical Research, London School of Hygiene and Tropical Medicine, London, United Kingdom; 2 Mycobacterial Research Laboratory, Anandaban Hospital, Kathmandu, Nepal; Hebrew University-Hadassah Medical School, Israel

## Abstract

**Background:**

Leprosy, a chronic granulomatous disease affecting the skin and nerves, is caused by *Mycobacterium leprae (M. leprae)*. The type of leprosy developed depends upon the host immune response. Type 1 reactions (T1Rs), that complicate borderline and lepromatous leprosy, are due to an increase in cell-mediated immunity and manifest as nerve damage and skin inflammation. Owing to the increase in inflammation in the skin of patients with T1Rs, we sought to investigate the activation of the innate immune system during reactionary events. Specifically, we investigated the expression levels of human beta-defensins (hBDs) 2 and 3 in the skin of patients with T1Rs, in keratinocytes, and in macrophages stimulated with *M. leprae* and corticosteroids.

**Results:**

Skin biopsies from twenty-three patients with Type 1 reactions were found to have higher transcript levels of hBD3 as compared to fifteen leprosy patients without Type 1 reactions, as measured by qPCR. Moreover, we observed that keratinocytes but not macrophages up-regulated hBD2 and hBD3 in response to *M. leprae* stimulation *in vitro*. Corticosteroid treatment of patients with T1Rs caused a suppression of hBD2 and hBD3 in skin biopsies, as measured by qPCR. *In vitro*, corticosteroids suppressed *M. leprae*-dependent induction of hBD2 and hBD3 in keratinocytes.

**Conclusions:**

This study demonstrates that hBD3 is induced in leprosy Type 1 Reactions and suppressed by corticosteroids. Furthermore, our findings demonstrate that keratinocytes are responsive to *M. leprae* and lend support for additional studies on keratinocyte innate immunity in leprosy and T1Rs.

**Trial Registration:**

Controlled-Trials.com ISRCTN31894035

## Author Summary

Leprosy, caused by *Mycobacterium leprae (M. leprae)*, is a chronic infection leading to potentially debilitating nerve damage. Although the infection is curable with multi-drug therapy, many patients continue to suffer from episodes of inflammation, called Type 1 reactions. These reactions may lead to nerve damage and deformation. Patients with Type 1 reactions are treated with corticosteroids to reduce the inflammation. Elucidating the immune factors contributing to leprosy Type 1 reactions will further clarify why many leprosy patients develop nerve and skin inflammation. We conducted a study to understand whether the antimicrobial peptides, human beta-defensins (hBD) 2 and 3, are increased in the skin of leprosy patients with Type 1 reactions. We also investigated keratinocyte expression of hBD2 and hBD3 in response to *M. leprae*. In this study, we found that hBD3 was significantly increased in the skin of leprosy patients with Type 1 reactions. Moreover, we demonstrated that corticosteroid treatment reduced the expression of hBD3 in patients with Type 1 reactions. We also found that keratinocytes incubated with *M. leprae* upregulated hBD2 and hBD3 *in vitro*; corticosteroids were found to suppress this response. These studies suggest a role for the innate immune system in leprosy Type 1 reactions.

## Introduction

Leprosy, caused by the pathogen *Mycobacterium leprae* (*M. leprae*), is a chronic granulomatous infection that causes skin lesions and potentially disabling neuropathy. Whilst the widespread use of multi-drug therapy (MDT) has reduced the disease burden globally, 213,036 new cases were reported in 2009 and leprosy continues to be an important health concern especially in areas such as India, Brazil, and Africa [1].

The clinical manifestations and classification of leprosy correlate with the type of immune response generated by the host. Tuberculoid leprosy (TT), on one pole of the spectrum, results from patients having an intense cell-mediated immune response and is characterized by an abundance of Th1 cytokines. On the opposite end of the spectrum are the patients with lepromatous leprosy (LL), characterized by a predominant humoral response and lack of *M. leprae*-specific cell-mediated immunity [2]. Patients with the borderline forms of leprosy fall between the two poles (TT and LL), are immunologically unstable, and are more prone to complicating reactions.

Type 1 reactions (T1Rs) correlated with an increase in Th1-mediated immunity while Type 2 reactions (also known as erythema nodosum leprosum, ENL) are associated with Th2 humoral complications. Reactions can occur in up to 30–50% of leprosy patients as single or recurrent episodes anytime before, during or years after effective multi-drug therapy (MDT). Due to the lipid content of the cell wall, *M. leprae* infection can leave behind persistent antigens within affected skin and nerve tissues rendering patients vulnerable to unpredictable leprosy reactions years after technical cure. Leprosy reactions, therefore, are a major factor of permanent neuropathy and disability development for patients. In response to the upgrades in cell-mediated immunity, T1Rs are often treated using oral corticosteroids, usually prednisolone. One of the effects of corticosteroids is the suppression of skin inflammation (edema and erythema) associated with cutaneous T1Rs.

Previous studies have suggested a prominent role for toll-like receptor (TLR) 2 in susceptibility to infection by *M. leprae* and *Mycobacterium tuberculosis* (Mtb). TLR2 is a receptor that recognizes pathogen associated molecular patterns (PAMPs); more specifically, these are diacylated and triacylated lipopetides that respectively bind to the TLR2/6 and TLR2/1 heterodimeric complexes [3]. *Mycobacteria* produce triacylated lipopetides and thereby induce immunological responses through the TLR2/1 complex [4]. TLR2/1 activation leads to the clearance of pathogens, such as *M. leprae* or Mtb, by macrophages, neutrophils, and epithelial cells. Several consequences of TLR2/1 activation are the production of pro-inflammatory cytokines and antimicrobial peptides. In particular, TLR2 stimulation leads to induction of cathelicidin, and human beta-defensins (hBDs) 2 and 3 in the epithelium and macrophages [5]. These antimicrobial peptides serve as the first line of defense against many bacteria, viruses, and fungi [6], [7], [8], [9]. In addition to directly killing pathogens, hBD2 induces cytokines including IL-6, IL-8, and IL-10 in monocytic cells [10].

Cell-specific studies on leprosy have predominantly focused on dermal cells such as macrophages, neutrophils, and T cells. In the dermis, macrophages are important cell types that promote Th1 responses. Macrophages are able to phagocytose and sequester viable *M. leprae* in their phago-lysosomes [11]. In the epidermis, keratinocytes are known to sense and eliminate pathogens by activating TLR-mediated innate responses while also affecting the dermal innate and adaptive immune responses. Although keratinocytes have not been a focus in leprosy, electron microscopy studies have in fact demonstrated *M. leprae* inside keratinocytes [12]. Other studies have also demonstrated that *M. leprae* proteins promote entry in keratinocytes [13] and induce MHCII in keratinocytes [14]. These data suggest an important role for keratinocytes in leprosy, as in other cutaneous infections. Specifically, the antimicrobial role of keratinocytes in defense has been previously demonstrated for cutaneous pathogens [15] but is still being understood in leprosy.

As *M. leprae* cannot be grown *in vitro* and animal studies are logistically challenging, genetic and *in vitro* infection studies have been employed to understand the role of host immunological factors in susceptibility to leprosy. One study demonstrates that *M. leprae* activates the TLR2/1 heterodimeric complex in HEK 293 cells, and activation is enhanced by Th1 cytokines [4]. Another study demonstrates that polymorphisms in hBD1 are associated with lepromatous leprosy [16]. Other studies conclude that a TLR2 mutation is associated with susceptibility to lepromatous leprosy [17], [18] and Type 1 reactions [19]. In support of the TLR2 polymorphic and mutation studies, *in vitro* mechanistic studies suggest that a TLR2 mutation leads to attenuated production of Th1 cytokines [20], [21]. Although these genetic studies are interesting and support an association of TLR2 in susceptibility to leprosy and Type 1 reactions, they have few subjects. Furthermore, the role of TLR2 activation in keratinocytes in Type 1 reactions has not yet been fully elucidated.

Several other studies on TLRs, surface proteins, and mycobacterial lipids have been performed that further suggest the importance of innate immunity in leprosy pathogenesis. TLR9 has been shown to mediate responses to mycobacterium [22], [23]. Genetic studies also indicate that TLR4 and NOD2 polymorphisms are associated with susceptibility to leprosy [24]. In addition, CD209 (DC-SIGN), a C-type lectin, allows for phagocytosis of *M. leprae*. This study further shows that the cytokine environment of tuberculoid leprosy (IL-15) leads to an induction of an antimicrobial program in macrophages in tuberculoid lesions [25]. Collectively, these studies demonstrate that the immune response in leprosy is both complex and diverse.

Here, we investigate the expression of hBD2 and hBD3 in the skin of leprosy patients with Type 1 reactions using qPCR. These skin biopsies were taken as part of a previous clinical trial on corticosteroids [26]. Furthermore, we determine the expression hBDs in *M. leprae*- treated keratinocytes and macrophages *in vitro* using qPCR and immunoflourescence. Finally, we aim to investigate whether corticosteroids suppress hBD expression in the skin biopsies as well as in keratinocytes *in vitro*. These studies will ideally lead to a greater understanding of the role of keratinocytes in leprosy, in diagnostics, and in susceptibility to infection by *M. leprae*.

## Materials and Methods

### Ethics statement

This study was approved by the Nepal Health Research Council and the Ethics Committee of the London School of Hygiene and Tropical Medicine (Number 4022). All subjects provided informed written consent.

### Skin biopsies

Full-thickness skin biopsies were taken from leprosy patients with and without Type 1 Reactions at Anandaban Hospital in Kathmandu, Nepal [26]. The case definitions and Ridley-Joplin classifications for the patients with leprosy and Type 1 reactions in this study have been previously published [26]. In a double blind parallel-group randomized controlled trial (RCT), patients with Type 1 reactions or nerve function impairment were randomized to receive high dose intravenous methylprednisolone (MP) followed by oral prednisolone or intravenous normal saline followed by oral prednisolone. Control subjects were untreated newly diagnosed leprosy patients who presented to Anandaban Hospital during the period of recruitment and follow-up of the methylprednisolone study. Biopsies from control subjects and T1Rs subjects were stored in RNA *Later* (Sigma) until processing. This study utilizes biopsies taken at day 0 (before corticosteroid treatment) and day 113 (at the end of corticosteroid treatment). We analyzed skin biopsies from 28 patients with T1Rs and 15 patients without T1Rs that served as controls. Of these 28 patients with T1Rs, 23 patients received corticosteroid treatment (oral prednisolone ± intravenous methylprednisolone). Skin biopsies of these 23 patients (with T1Rs and receiving corticosteroid treatment) were analyzed on day 0 and day 113, before and after corticosteroid treatment, respectively. Cellular infiltration, the relative abundance of infiltrated cells in the dermis (whose nuclei stain with hematoxylin), was performed as previously described [27].

### Cell culture

A human keratinocyte cell line, (HaCaT cells), kindly supplied by Dr. Edel O'Toole (St. Barts and The London), were maintained in DMEM with 10% (v/v) heat-inactivated fetal calf serum (FCS, Fisher scientific), 100 µg/ml (100 IU) penicillin/streptomycin, and 2 mM L-glutamine on T75 ml flasks at 37°C at 5% CO_2_. At 80% confluency, cells were lifted using Accutase (Sigma) and reseeded at a 1∶10 ratio. For stimulation assays, HaCaT cells were seeded into 12-well plates and at 50% confluency, were stimulated for 24 hours with whole cell sonicated *M. leprae* (supplied by Colorado State University) at 1 µg/ml, 10 µg/ml, 100 µg/ml or prednisolone (Sigma) at 1 nM, 10 nM, or 100 nM or 1 µM Pam3CSK4 (Toll-like receptor 2/1 ligand). Assays were performed 3 times in triplicate.

To obtain primary human macrophages, peripheral blood mononuclear cells (PBMCs) were first isolated from 50 ml whole blood using a Ficoll gradient. PBMCs were cultured in RPMI with 10% FCS on a T75 flask for 24 hours at 37°C to allow monocyte adherence. Nonadherent cells were gently removed with warm media, adherent monocytes were washed with PBS and cultured for 48 hours. Cells were lifted with Accutase, washed, and re-cultured in 12 well plates for 48 hours at 10∧6 cells/ml. Stimulation assays were performed as above. Assays were performed 2 times in triplicate.

### RNA isolation

After HaCaT and macrophage stimulation, RNA was isolated using TRIzol Reagent (Invitrogen). According to manufacturer's instructions, 0.5 µg of total RNA was used for cDNA synthesis by the iSCRIPT cDNA synthesis kit (Bio-Rad). For the skin biopsies, RNA was isolated by first disrupting the skin using disposable pestles and lysis buffer from the RNeasy Fibrous Tissue Mini Kit. The skin was subjected to a Qiashredder (Qiagen) and RNA was isolated using the RNeasy Fibrous Tissue Mini Kit (Qiagen) per manufacturer's instructions. RNA concentration was ascertained using Nanodrop (ND-1000). RNA was converted to cDNA using the Iscript kit (Invitrogen) per manufacturer's instructions.

### Real-time quantitative RT-PCR

Real-time quantitative RT-PCR was performed on an ABI 7900HT Fast Real-Time PCR (ABI) using Power SybrGreen (ABI) and primers at a final concentration of 0.2 µM. The primer sequences are as follows: 36B4 F: 5′-TCGAACACCTGCTGGATGAC, 36B4 R: 5′-CCACGCTGCTGAACATGCT, DEFB4 (hBD2) F: 5′-GGTGTTTTTGGTGGTATAGGC, DEFB4 (hBD2) R: 5′-AGGGCAAAAGACTGGATGACA, DEFB103 (hBD3) F: 5′-GCTGCCTTCCAAAGGAGGA, DEFB103 (hBD3) R: 5′-TTCTTCGGCAGCATTTTCG, TLR2 F: 5′-CATTCCCTCAGGGCTCACAG, TLR2 R: 5′-TTGTTGGACAGGTCAAGGCTT, TLR1 F: 5′-GAGGCAATGCTGCTGTTCAG, TLR1 R: 5′-CCTGGTACCCCTATTAGTGTT. The ΔΔC_T_ method was used for quantification of gene expression. The target genes were normalized to the endogenous reference gene that encodes an acidic ribosomal phosphoprotein P0 (RPLP0) also known as 36B4. Target genes are reported as the fold difference relative to the reference gene. All of the stimulation experiments were performed in triplicate and repeated at least two times. All of the experiments using skin biopsies were analyzed by real-time PCR in triplicate.

### Immunoflorescence

HaCaT cells were seeded into chamber slides and stimulated at 50% confluency with 1 µM Pam3CSK4, 100 µg/ml *M. leprae* whole cell sonicate, or 100 µg/ml prednisolone as indicated. After 24 hours, the supernatant was removed and cells were fixed with acetone for 15 minutes at room temperature. The slides were subsequently washed 3 times with 1× phosphate-buffered saline (PBS), blocked for 1 hour at room temperature with 3% bovine serum albumin in 1× PBS, and stained with primary antibodies rabbit anti-hBD3 (Abcam) or rabbit IgG in 3% bovine serum albumin/1× PBS overnight at 4°C. After washing the cells 3 times with PBS, the cells were stained with secondary antibodies goat anti-rabbit IgG (Sigma), anti-rabbit IgG in 3% bovine serum albumin in 1× PBS for 1 hour at room temperature. Slides were washed three times in 1× PBS and were mounted in ProLong Anti-Fade reagent (Molecular Probes).

### Statistical methods

All statistical analyses were performed using GraphPad Prism 4.0. An unpaired t-test was used when appropriate. Values of *p*<0.05 were considered significant.

## Results

### Human beta-defensin 3 is up-regulated in cutaneous leprosy type 1 reactions

**Figure 1:**
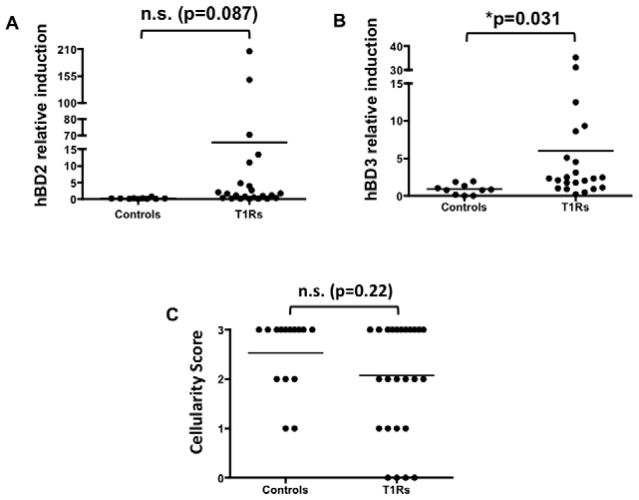
Human beta-defensin 3 is up-regulated in the skin of leprosy patients with Type 1 reactions. Skin biopsies from leprosy patients with Type 1 reactions (T1Rs) and leprosy patients without T1Rs (Controls) were assessed for their expression hBD2 and hBD3 as measured by qPCR. The skin of patients with T1Rs showed non-significantly elevated (A) hBD2 expression and a significant increase in (B) hBD3 expression. (C) Cellular infiltration in the T1Rs and the Controls show no significant differences. For the statistical analyses, values were first converted to a Gaussian distribution by taking their square and subsequently an un-paired t-test was performed. *p<0.05 is considered significant.

To determine the levels of hBD2 and hBD3 expression in Type 1 reactions, the relative stable transcript levels were assessed in skin biopsies of control leprosy patients and leprosy patients with Type 1 reactions. We found a trend towards increased hBD2 expression in the skin of patients with Type 1 reactions by 16.6 fold-change, though it was not statistically significant (p = 0.087) (Figure 1A). Moreover, we found that hBD3 was significantly up-regulated in the skin of patients with Type 1 reactions by 5.96 fold-change (p<0.05) (Figure 1B).

Since T1Rs are clinically characterized by edema and erythema, we investigated whether the increased hBD expression was due to an increase in cellular infiltration. We found that the cellularity score of the controls and T1Rs are similar (Figure 1C). These data demonstrate that the increase in hBD2 and hBD3 expression is due to activation of the innate immune response rather than due to an increase in the number hBD-expressing cells in T1Rs.

### *Mycobacterium leprae* induces human beta-defensins in keratinocytes but not macrophages *in vitro*

**Figure 2:**
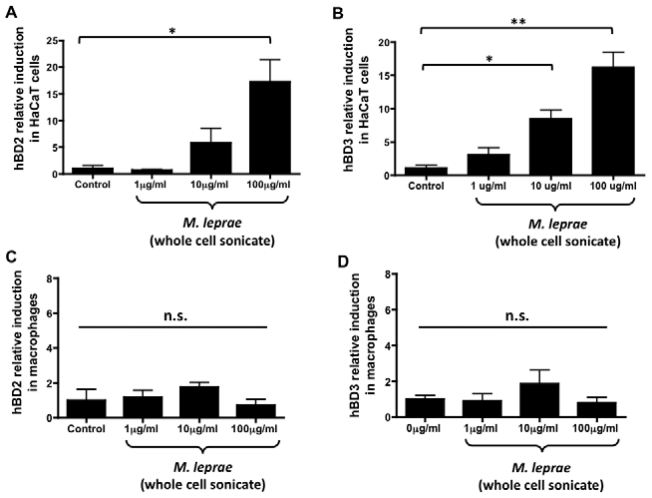
*Mycobacterium leprae* induces human beta-defensins 2 and 3 in keratinocytes but not macrophages. To determine whether keratinocytes would induce an antimicrobial program to *M. leprae*, immortalized keratinocytes (HaCaT cells) were stimulated with *M. leprae* whole cell sonicate at 1 µg/ml, 10 µg/ml, 100 µg/ml for 24 hours. *M. leprae* induced a dose-dependent up-regulation of hBD2 (A) and hBD3 (B) stable transcript levels as measured by qPCR. Macrophages cultured with *M. leprae* whole cell sonicate at 1 µg/ml, 10 µg/ml, 100 µg/ml for 24 hours showed no significant induction if hBD2 (C) and hBD3 (D). Experiments were performed at least twice in triplicate. **p<0.01, *p<0.05, n.s. (no significance) were determined by an unpaired t-test when compared to the controls.

Since the skin is comprised of multiple cell types that play a role in cutaneous innate immunity, we sought to determine in which cell type the hBDs are being up-regulated. Keratinocytes, the primary cell in the epidermis, were first investigated for their ability to up-regulate the hBDs in response to *M. leprae*. We found that HaCaT cells, immortalized keratinocytes, increased their expression of hBD2 and hBD3 in a dose-dependent manner in response to *M. leprae* whole cell sonicate (Figure 2A and 2B). To determine if macrophages induce a similar antimicrobial program to HaCaT cells, primary macrophages were stimulated with *M. leprae* whole cell sonicate at the indicated concentrations for 24 hours. We found that HaCaT cells but not macrophages up-regulate hBD2 and hBD3 in response to *M. leprae* (Figure 2C and 2D). We also attempted to stain skin biopsies from patients with Type 1 reactions and from control leprosy patients with two different anti-hBD3 antibodies. Unfortunately, both antibodies exhibited non-specific staining and could not be used to assess the temporal or relative quantity of hBD3 in the biopsies (data not shown).

**Figure 3:**
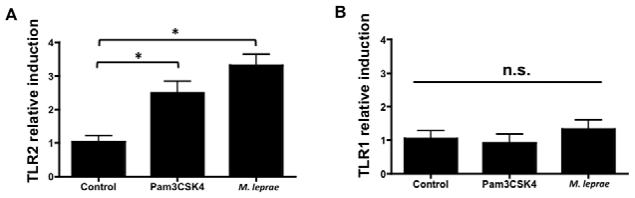
*Mycobacterium leprae* induces TLR2 but not TLR1 in keratinocytes. Since activation of the TLR2/1 complex leads to keratinocytes expression of hBD2 and hBD3, increase in TLR2 and/or TLR1 may similarly lead to increased hBD expression. To evaluate whether the expression of TLR2 and TLR1 are induced by *M. leprae*, HaCaT cells were stimulated with 1 nM of the TLR2/1 agonist Pam3CSK4 or 100 µg/ml *M. leprae* for 24 hours. Both Pam3CSK4 and *M. leprae* significantly up-regulated stable transcript levels of (A) TLR2 but not (B) TLR1 in HaCaT cells. *p<0.05 or n.s. (no significance) were determined by an unpaired t-test when compared to the controls.

Since hBD production likely results from TLR2/1 activation by *M. leprae*, we investigated whether TLR2 and TLR1 are induced in *M. leprae* or TLR2/1 agonist (Pam3CSK4)-treated keratinocytes. We found that *M. leprae*, as well as synthetic triacylated-lipopetide Pam3CSK4, induce TLR2 but not TLR1 stable transcript levels (Figure 3A and 3B). Taken together, these data demonstrate that *M. leprae* activates keratinocytes through up-regulating the human beta-defensins and TLR2 *in vitro*.

### Corticosteroids suppress human beta-defensin expression in the skin of patients with Type 1 reactions

**Figure 4:**
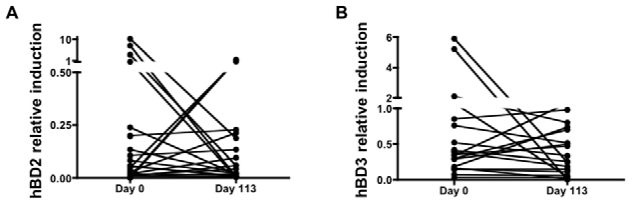
Corticosteroids suppress human beta-defensins 2 and 3 in leprosy patients with Type 1 reactions. The expression of hBD2 and hBD3 were assessed in skin biopsies of leprosy patients with Type 1 reactions before (day 0) and after (day 113) corticosteroid treatment. For hBD2, 13 out of 23 patients demonstrated suppression, with a mean transcript suppression of 78.5% when comparing day 113 to day 0 (A). For hBD3, 16 out of 23 patients demonstrated suppression, with a mean transcript suppression of 61.3% when comparing day 113 to day 0 (B).

To determine if oral corticosteroid treatment suppresses the innate immune response in leprosy patients with Type 1 reactions, the expression of hBD2 and hBD3 was evaluated from the skin of patients at day 0 and at day 113 of corticosteroid treatment. For hBD2, we observe a mean relative transcript suppression of 78.5% with 13/23 (56.5%) patients showing a decrease in hBD2 expression (Figure 4A). For hBD3, we observe a mean relative transcript suppression of 61.3% with 16/23 (69.6%) patients showing a decrease in hBD3 expression (Figure 4B). In addition, 14 of the 23 patients with Type 1 reactions received an initial dose of intravenous methylprednisolone before a course of oral prednisolone. We found that methylprednisolone caused no additional suppressive effect on hBD2 and hBD3 and in fact, the two patient groups were nearly identical in regards to their cutaneous suppression of hBD2 and hBD3 (data not shown). Thus, all patients receiving corticosteroid (prednisolone and methylprednisolone) were grouped together for collective analyses (Figures 4A and 4B). These data illustrate that oral corticosteroids suppress hBD2 and hBD3 in the skin of patients with Type 1 reactions.

### Keratinocyte induction of human beta-defensins by *M. leprae* is suppressed by corticosteroids *in vitro*

**Figure 5:**
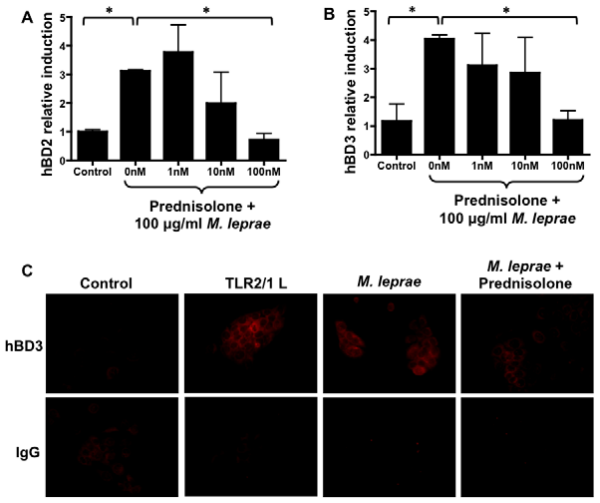
Prednisolone suppresses keratinocyte up-regulation of human beta-defensins 2 and 3 by *M. leprae in vitro*. To determine whether prednisone suppresses keratinocyte expression of hBD2 and hBD3, HaCaT cells were stimulated with 100 µg/ml *M. leprae* and 0 (Control), 1, 10, and 100 nM of prednisone for 24 hours. Prednisolone caused a dose-dependent suppression hBD2 (A) and hBD3 (B). To confirm the suppression of hBD3 by prednisolone on the protein level, HaCaT cells were cultured in chamber slides with media alone (Control), 1 nM Pam3CSK4, 100 µg/ml *M. leprae*, or 100 µg/ml *M. leprae* and 100 nM prednisolone. Strong intracellular hBD3 expression is observed in cells treated with Pam3CSK4 or *M. leprae*. Prednisolone reduces the level of intracellular hBD3 produced by HaCaTs in response to *M. leprae* (C). IgG controls are represented by the bottom panel images. Significance (*p<0.05) was determined by an unpaired t-test.

We have demonstrated in Figure 2 that keratinocytes but not macrophages up-regulate hBD2 and hBD3 in response to *M. leprae* antigens, suggesting a role for the epidermidis in T1Rs. Since corticosteroid treatment resulted in a suppression of the hBDs in the skin of patients, we sought to determine if corticosteroids would similarly result in the suppression of hBDs in cultured keratinocytes. To determine if corticosteroids suppress keratinocyte innate immunity, HaCaT cells were stimulated *in vitro* with *M. leprae* whole cell sonicate and an increasing dose of prednisolone. We found that prednisolone significantly suppresses hBD2 and hBD3 induction by *M. leprae* whole cell sonicate (Figure 5A and 5B). Suppression by prednisolone also appears to occur in a dose-dependent manner (Figure 5A and 5B).

To confirm that prednisolone suppresses the protein levels of hBD3 in keratinocytes, immunofluorescence was performed on HaCaT cells. We found that the TLR2/1 L and *M. leprae*-treated cells have an increase in intracellular hBD3 while prednisolone reduces the intracellular level of hBD3 induced by *M. leprae* (Figure 5C). Taken together, these data demonstrate that prednisolone directly suppresses *M. leprae*-dependent hBD3 induction in keratinocytes.

## Discussion

In this study, we have demonstrated that during Type 1 reactions, hBD3 is significantly up-regulated in the skin. This up-regulation appears to be due to an increase in gene expression rather than an increase in cellular infiltration (Figure 1). In addition, we found that keratinocytes but not macrophages up-regulate hBD2 and hBD3 in response to *M. leprae in vitro*. This result was unanticipated since macrophages are known to play a major role in leprosy pathogenesis. Moreover, we find it interesting that keratinocytes were robustly responsive to *M. leprae*.

For these *in vitro* experiments, nonviable, sonicated *M. leprae* was employed. While the possibility cannot be ruled out that live infection may generate immune variation, leprosy reactions appear to occur or persist regardless of *M. leprae* viability [28]. In addition, most patients receiving corticosteroids for leprosy reactions are concurrently receiving MDT or have completed MDT, rendering *M. leprae* essentially nonviable within the study time period [29]. Yet, in the future, viable *M. leprae* could be useful to assess the host response during an active infection.

Few previous studies have suggested that the epidermis plays a role in leprosy or reactions, in spite of the cutaneous manifestations. This may be attributed to the fact that keratinocytes are often thought of as part of the barrier rather than as an immune organ. In fact, keratinocytes play a significant role in immune homeostasis and innate immunity. Our data demonstrating that HaCaTs up-regulated TLR2 in response to *M. leprae* suggests that the cells are preparing to respond to pathogens. The downstream effect of increased TLR2 expression would likely lead to an enhanced ability to sense lipo-polypeptides and subsequent up-regulation of antimicrobial peptides.

There have been a number of important studies on the cytokine milieu in leprosy [2], [30]. It has also been demonstrated that proinflammatory cytokines induce the expression of antimicrobial peptides in gingival keratinocytes [31]. It is possible that the cytokine environment is causing an induction of hBDs, rather than direct mycobacterial interaction with keratinocytes. Yet, mycobacterial lipids are known to directly induce TLR-mediated antimicrobial responses. For leprosy, electron microscopy studies demonstrating *M. leprae* inhabitance in keratinocytes further suggest a direct interaction. Finally, recent studies on cutaneous microbiota demonstrate that an intricate relationship exists between bacterium and keratinocytes [32], [33], [34]. In the future, it would be interesting to elucidate the specific cellular and molecular interactions between keratinocytes and *M. leprae*.

The effects of steroids on the expression of hBDs were not surprising, yet we included these studies to correlate the hBD expression with T1Rs. Corticosteroids that bind the glucocorticoid receptor have been shown to suppress the expression of proinflammatory cytokines through NFκB [35]. Toll-like receptor 2 initially signals through MyD88 and IRAK, subsequently leading to NFκB activation and production of hBDs [36]. Thus, TLR2-mediated suppression of hBDs by steroids is expected but also confirms that steroids directly affect the dermis and epidermis. It would be interesting to determine the status of keratinocytes in the skin of patients with T1Rs in the presence and the absence of steroids. These studies would further clarify the relative importance of keratinocytes in T1Rs.

Based on our data, we hypothesize that the epidermis plays a more substantial role than previously thought. Many studies have demonstrated that keratinocytes can kill bacteria, viruses, fungi, and parasites through the production of antimicrobial peptides. We hypothesize that the up-regulation of hBDs in keratinocytes leads to reduction of *M. leprae* during Type 1 reactions for several reasons. First, since the hBDs are directly antimicrobial, they may directly kill the *M. leprae* living in the keratinocytes or may be secreted and kill the bacteria in the upper papillary dermis near the basement membrane. Unfortunately, as we are currently unable to grow *M. leprae in vitro*, we are consequently not able to conclusively test the antimicrobial role of the hBDs without a mouse footpad or an armadillo model of infection. Another means by which the hBDs may lead to the reduction of *M. leprae*, which occurs during Type 1 reactions, is due to their pro-inflammatory potential. If the basal keratinocytes release the hBDs into the dermis, they may activate dermal macrophages, subsequently leading to cytokine production and an antimicrobial response. An increase in Th1 cytokines will activate macrophages, leading to a further reduction of *M. leprae*. It would be interesting to determine whether defensins promote the differentiation of macrophages in Type 1 reactions.

Additional experiments, such as immunohistochemistry of skin sections for the hBDs and TLR2 knockdown experiments, would be useful to more conclusively demonstrate mechanisms by which the innate immune system is activated.

In conclusion, we show that hBD3 is upregulated in leprosy Type 1 reactions. Elucidating the means by which keratinocytes interact with *M. leprae* and cells in the dermis will shed light on future diagnostics, host-pathogen interplay, and mechanisms of infection.
